
JOURNAL INFORMATION
==============================
NLM Title Abbreviation: Inter Econ
Journal ID: Inter Econ
NLM Journal ID: 101086399

Inter Economics

ISSN: 0020-5346

ARTICLE INFORMATION
==============================
PMCID: PMC8021642
DOI: 10.1007/s10272-021-0954-0
Article ID: 954
Article version: 1
Subjects: Forum Introduction

European Integration and National Elections After COVID-19

Electronic publication date: 2021 Apr 6
Print publication date: 2021
Volume: 56
Issue: 2
First page: 64
Last page: 64
Copyright: © The Author(s) 2021
License: Open Access: This article is distributed under the terms of the Creative Commons Attribution 4.0 International License (https://creativecommons.org/licenses/by/4.0/).
Open Access funding provided by ZBW — Leibniz Information Centre for Economics.
License URL: https://creativecommons.org/licenses/by/4.0/

issue-copyright-statement: © The Author(s) 2021

==============================
DOI: 10.1007/s10272-021-0954-0

### European Integration and National Elections After COVID-19

**The United Kingdom's departure from the European Union and the COVID-19 crisis are offering a window of opportunity for a deeper fiscal union and further European integration in various policy areas including foreign affairs and defence, tax harmonisation and climate change. While this debate goes far beyond national boundaries, the voters who cast their ballots are still as national as ever. When the EU emerges from the pandemic, the political landscape in different member states will likely become even more crucial for European integration. In this context, the recent elections in the Netherlands, the latest government crisis in Italy and the upcoming national elections in Germany, France and Hungary might show whether that window of opportunity remains. This Forum provides insight into how the domestic politics of EU member states pose a challenge to, and an opportunity for, the future of the European integration project.**

### Europe After COVID-19: A New Role for German Leadership?

**Francesco Saraceno**, OFCE – Sciences Po, Paris, France; and Luiss, Rome, Italy.

### The EU Elephant: Europe in the 2021 Dutch General Elections

**Simon Otjes**, Leiden University; and Groningen University, Netherlands.

### Presidential Election 2022: A Euroclash Between a "Liberal" and a "Neo-Nationalist" France Is Coming

**Thierry Chopin**, Université Catholique de Lille, ESPOL, France.

**Samuel B. H. Faure**, Sciences Po Saint-Germain-en-Laye, France.

### Italy's Political Turmoil and Mario Draghi's European Challenges

**Mario Pianta**, Scuola Normale Superiore, Florence, Italy.

### Europhile Public vs Eurosceptic Governing Elite in Hungary?

**Borbála Göncz**, Corvinus University of Budapest, Hungary.

**György Lengyel**, Corvinus University of Budapest, Hungary.
